# One-pot synthesis of isosorbide from cellulose or lignocellulosic biomass: a challenge?

**DOI:** 10.3762/bjoc.16.143

**Published:** 2020-07-16

**Authors:** Isaline Bonnin, Raphaël Mereau, Thierry Tassaing, Karine De Oliveira Vigier

**Affiliations:** 1 Université de Poitiers, IC2MP, UMR CNRS 7285, 1 rue Marcel Doré, 86073 Poitiers Cedex 9, France; 2 Institut des Sciences Moléculaires, UMR 5255 CNRS-Université de Bordeaux, 351 Cours de la Libération, 33405 Talence Cedex, France

**Keywords:** catalysis, cellulose, isosorbide, lignocellulosic biomass

## Abstract

The catalytic conversion of (ligno)cellulose is currently subject of intense research. Isosorbide is one of the interesting products that can be produced from (ligno)cellulose as it can be used for the synthesis of a wide range of pharmaceuticals, chemicals, and polymers. Isosorbide is obtained after the hydrolysis of cellulose to glucose, followed by the hydrogenation of glucose to sorbitol that is then dehydrated to isosorbide. The one-pot process requires an acid and a hydrogenation catalyst. Several parameters are of importance during the direct conversion of (ligno)cellulose such as the acidity, the crystallinity and the particle size of cellulose as well as the nature of the feedstocks. This review highlights all these parameters and all the strategies employed to produce isosorbide from (ligno)cellulose in a one-pot process.

## Introduction

**Scheme 1:**
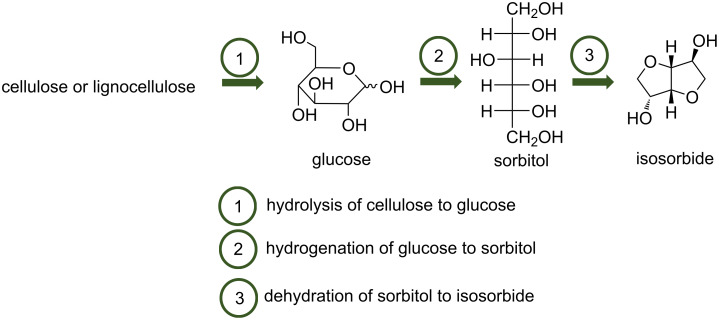
Conversion of cellulose to isosorbide.

Cellulose, a homopolymer of ᴅ-glucose, is the most abundant component of lignocellulosic biomass. Cellulose is a crystalline polymer due to its intra- and intermolecular hydrogen bond network. The conversion of cellulose to added value chemicals has received a lot of interest due to the rarefaction of fossil oil and environmental concerns. One of the interesting reactions is the conversion of cellulose to isosorbide, a 1,4:3,6-dianhydrohexitol [1]. This reaction occurs in several steps: 1) hydrolysis of cellulose to glucose 2) hydrogenation of glucose to sorbitol and 3) dehydration of sorbitol to isosorbide (Scheme 1).

Isosorbide, a molecule obtained from biomass can find many applications such as additives, pharmaceuticals [2–3] and monomers for polymer industries [4–6]. For instance, one polymer obtained from isosorbide, poly(ethylene-*co*-isosorbide) terephthalate, can replace polyethylene terephthalate (PET) [7]. In the production of polycarbonate and epoxy resins, the physicochemical properties of isosorbide allow the replacement of bisphenol A by this bio-based molecule [8–9]. At an industrial level, isosorbide is produced from the double dehydration of ᴅ-sorbitol using a strong acid catalyst [10–11]. ᴅ-Sorbitol is produced from the hydrogenation of glucose obtained mostly from the hydrolysis of starch, but also from sucrose or cellulose. Consequently, the cellulose valorization can be realized from the one-pot conversion of cellulose to isosorbide. The hydrolysis of cellulose to glucose, the first step, is reported using acid catalysts such as H_3_PO_4_, H_2_SO_4_ and HCl as well as heterogeneous catalysts tungstolitic acid (H_4_SiW_12_O_40_), niobium phosphate, Amberlyst-70 and Dowex-H [1,12]. For the second step (hydrogenation of glucose to sorbitol), the common catalysts used are Ru- and Ni-based ones [13–16]. Then sorbitol is dehydrated trough an acid-catalyzed process leading to isosorbide with the formation of 1,4- and 3,6-sorbitan intermediates in the third step [17]. The synthesis of isosorbide is performed under high hydrogen pressures and high temperatures to allow efficient hydrogenation of glucose and dehydration of sorbitol and sorbitans [12,18]. In 2010, Almeida et al. studied each step for the synthesis of isosorbide from cellulose using molten ZnCl_2_ associated with different catalysts [19]. Based on the study of each step reported in the literature, several researchers investigated the direct conversion of cellulose or lignocellulosic biomass to isosorbide. Several strategies were employed such as a combination of homogeneous acid and supported metal catalyst, or a combination of supported metal catalyst and solid acid or a metal on an acid support. Here, we will report all these strategies to perform the one-pot conversion of (ligno)cellulose to isosorbide and the key parameters of this reaction (acidity, nature of the feedstocks, poisoning of the catalyst).

## Review

### Combination of an acidic homogeneous catalyst and a supported metal catalyst

**Scheme 2:**
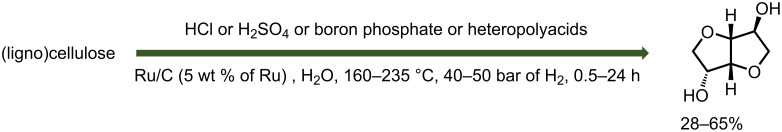
Combination of mineral acids or heteropolyacids and a supported metal catalyst to produce isosorbide from (ligno)cellulose.

The conversion of cellulose or lignocellulosic biomass to isosorbide was studied by combining a homogeneous acid catalyst to promote the hydrolysis of cellulose to glucose and the dehydration of sorbitol to isosorbide and a supported metal catalyst to hydrogenate glucose to sorbitol. Several homogeneous catalysts were used such as mineral acids, boron phosphate and heteropolyacids (Scheme 2).

Palkovits et al*.* studied the combination of supported noble metal catalysts based on Pt, Pd and Ru with dilute mineral acids such as phosphoric or sulfuric acid for the conversion of cellulose and even spruce in a one-step hydrogenolysis reactions to form C4 to C6 sugar alcohols [20]. For the C6 sugar alcohols including the isosorbide formation, the yield was below 6% whatever catalyst was used at 160 °C, under 50 bar of H_2_ in a 36 mL stainless steel autoclave equipped with Teflon inserts for a reaction time between 1 h to 5 h. Another study was performed using sulfuric or hydrochloric acid in the presence of Pt/C, Pd/C or Ru/C catalysts [21]. In the presence of Pt/C and Pd/C associated to HCl or H_2_SO_4_ the isosorbide yield was very low (below 4%) for a total conversion of cellulose in agreement with the results obtained by Palkovits et al. [20]. In the presence of Ru/C (5 wt % of Ru, 20 mg) associated with HCl (235 °C, 6.0 MPa of H_2_, 60 min, 10 mL of acidified water, 0.01 M of HCl, in a 50 mL Teflon-lined stainless steel autoclave), 41% of isosorbide was obtained for a full conversion of cellulose. If the Ru/C catalyst was associated with H_2_SO_4_ only 14% of isosorbide was produced under similar conditions. This study showed that the nature of the mineral acid and thus the acidity is of importance to produce isosorbide from cellulose. The most active metal catalyst is Ru/C among the metal-supported solids studied. One can mention, that by decreasing the reaction temperature from 235 °C to 215 °C, 50% of isosorbide was obtained after 30 min of reaction under 6.0 MPa of H_2_ in the presence of 20 mg of Ru/C (5 wt % of Ru) and HCl in 10 mL of water with a concentration of 0.01 M. A similar strategy was employed but starting from lignocellulosic biomass [22] such as bagasse pulp (BP) containing 95 wt % of cellulose and 5 wt % of lignin and the results were compared to glucose and microcrystalline cellulose (MCC). Commercial Ru/C combined with H_2_SO_4_ led to higher isosorbide yield (50% under optimized conditions, 220 °C under 40 bar of H_2_ for 2 h and 0.5 M H_2_SO_4_ (aq) 30 mL, 40 mg of Ru/C (5 wt % of Ru) in a glass insert in an autoclave) than Pt/C, Pd/C and Rh/C catalysts for the one-pot one step conversion of BP to isosorbide. The nature of the acids to catalyze the hydrolysis of cellulose and dehydration of sorbitol and sorbitans was studied and their efficiency decreased in the order of H_2_SO_4_ > triflic acid (HOTf) > trifluoroacetic acid (TFA) > Amberlyst-38 > HCl > HNO_3_. It was shown that when MCC was used as starting material, in the presence of Ru/C and H_2_SO_4_, 50% yield of isosorbide was obtained. These results are in contradiction with the results observed by Liang et al. [21]. Hence, a isosorbide yield of 14% was obtained. The reasons could be the different conditions used. In the work of Liang et al. [21], 0.2 g of cellulose was added in 10 mL of acidified water with a concentration of H_2_SO_4_ of 0.05 M in the presence of 20 mg of Ru/C at 235 °C whereas 0.19 g of cellulose was added in 30 mL of acidified water (H_2_SO_4_ concentration of 0.5 M, 10 times higher than in the other work) in the presence of 40 mg of Ru/C at 220 °C. All these parameters can explain the difference observed and again it confirms that the pH is a key parameter in the conversion of cellulose or lignocellulosic biomass to isosorbide.

**Scheme 3:**
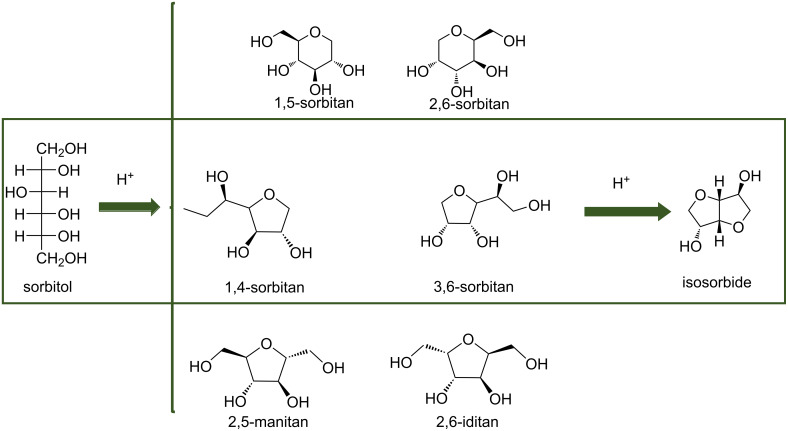
Conversion of sorbitol to isosorbide via the formation of sorbitans.

The main bottlenecks of this one-step process are the deactivation of the commercial Ru/C catalyst and the degradation of isosorbide upon prolonged reaction time. Keskivali et al. studied the two-step reaction [22]. The first step included the hydrolysis of cellulose to glucose, hydrogenation of glucose into sorbitol and partial dehydration of sorbitol to sorbitans. The second step was the dehydration of sorbitol and 1,4-sorbitan to isosorbide (Scheme 3). Optimal reaction conditions for the first step are middle temperatures to enhance the recyclability of Ru/C catalyst. 59% of sorbitol was obtained at 170 °C with 0.5 M of H_2_SO_4_, 20 bar of H_2_ for 2 h and in the presence of 0.02 mmol of Ru. The second step was performed starting from the first step hydrogenation solution after the removal of the Ru/C catalyst by filtration. A temperature higher than 200 °C was used in order to obtain a high isosorbide yield. A reaction temperature of 240 °C and 270 °C led to an isosorbide yield of 43% and 47%, respectively, after 1 h of reaction. A prolonged reaction time led to a decrease of the isosorbide yield due to side reactions. A decrease of the temperature from 240 °C to 170 °C led to an increase of the isosorbide yield (58%) after 24 h of reaction showing that a lower temperature can be used for this reaction but a prolonged reaction time is required.

The authors observed a deactivation of the Ru-catalyst in the presence of substrates containing lignin that was probably due to fouling and poisoning. Moreover, the presence of sulfuric acid could have been modified the Ru/C catalyst. With the increase of strong acid sites on the catalyst surface, the activity and recyclability of the catalyst was improved. A modification of the Ru/C hydrogenation catalyst’s surface by sulfonation and oxidative treatment was performed and had a significant effect on the catalyst properties in the isosorbide synthesis. Hence, strong acid sites are generated on the surface of the catalyst support leading to an increase of its hydrophilicity. The catalyst treated with a solution of 10 M of sulfuric acid during 3 h was recycled at least for four consecutive runs starting from bagasse pulp. Under optimized conditions (30 mL of aqueous solution of 0.5 M H_2_SO_4_; first step reaction conditions: 170 °C, 20 bar H_2_, 2 h and 0.02 mmol of modified Ru/C catalyst, 3 h; second step reaction conditions: 200 °C, 40 bar H_2_, 6 h after the removal of Ru/C catalyst) isosorbide was generated in high yields of 56–57% (49–50 wt %) from different cellulosic substrates. This study showed that the acidity is important in the conversion of lignocellulosic biomass to isosorbide and that the acid can react with the metal-supported catalyst.

Another study was devoted to the use of boron phosphate for the conversion of cellulose into liquid hydrocarbon C_1_–C_6_ over Ru/C [23]. A mixture of MCC (0.8 g) and Ru/C catalyst (5 wt % of Ru, 0.2 g) in 40 mL of water under 60 bar of H_2_ at 230 °C for 24 h in a 100 mL stainless-steel autoclave was used in the one-pot conversion of cellulose. C_1_–C_4_ compounds were obtained predominantly. However, with the addition of 4 mmol boric acid (H_3_BO_3_), which is a weak acid, sorbitol was observed with 5% yield along with 1% of sorbitan and 10% of isosorbide. The authors showed that a complex of borate-polyol and sorbitol or sorbitan as polyols was formed leading to an increase of their stabilities and thus their yields. When boric acid is replaced by phosphoric acid (which is a strong acid) for the conversion of cellulose, an isosorbide yield of 29% is reached indicating that double cyclodehydration is favored under these conditions. On the contrary, the addition of equimolar amounts of boric acid and phosphoric acid decreases the yield of isosorbide and sorbitan from 29% to 22% and from 5% to 3%, respectively. A similar tendency was observed in the presence of boron phosphate. This was due to the slow dissolution of boron phosphate in aqueous solution implying a release of H^+^ according to reaction time. They also observe an increase of isosorbide production from 18% to 28% with the increase of boron phosphate amount from 1 to 4 mmol due to the increase of the media acidity at 230 °C under 6.0 MPa of H_2_ in the presence of 0.2 g of Ru/C in 40 mL of water.

**Scheme 4:**
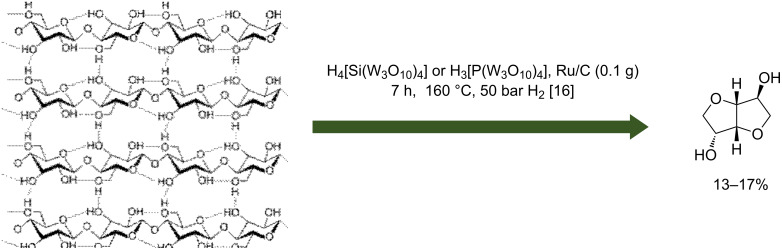
Conversion of cellulose to isosorbide in the presence of heteropolyacids and metal-supported catalyst [24].

An interesting family of acids was used in combination with metal-supported heteropolyacids as catalyst. Heteropolyacids were chosen since they are nontoxic or noncorrosive chemicals (Scheme 4).

Palkovits et al. showed that heteropolyacids (tungstosilic H_4_SiW_12_O_40_ or phosphotungstic acid and H_3_PW_12_O_40_) can be used in association with a Ru/C catalyst to produce isosorbide from α-cellulose [24]. The experiments were made in a batch reactor containing 500 mg of α-cellulose, 100 mg of 5 wt % Ru/C catalyst and 10 mL of water with an acid concentration from 3.47 to 55.1 mmol·L^−1^ at 160 °C under 50 bar of H_2_ (25 °C). It was reported that the rate of cellulose hydrogenolysis reaction depends on the acid concentration and reaction time. Indeed, an increase of isosorbide and sorbitan yields was observed after 7 h of reaction at 160 °C and 50 bar H_2_ with an acid concentration of 55.1 mmol·L^−1^. The isosorbide yield was 17% and 13% for a cellulose conversion above 90% for respectively H_4_[Si(W_3_O_10_)_4_] and H_3_[P(W_3_O_10_)_4_].

Later, Op de Beeck et al. explored a combination of Ru/C and tungstosilic acid (H_4_SiW_12_O_40_) for the one-pot conversion of cellulose into isosorbide [25]. They studied the different steps of the reaction. They have shown that for the last step (dehydration of sorbitol to isosorbide) the increase of the temperature from 190 to 230 °C and the acid concentration from 0 to 61 mM of H^+^ allows the dehydration of sorbitol to isosorbide (60% yield). However in this reaction, if the acid concentration is too high (over 61 mM of H^+^), polymeric compounds were formed limiting the yield to isosorbide. Based on these results, it is clear that a control of the acidity is required for the dehydration of sorbitol to isosorbide. Another important parameter is the control of the sorbitol concentration. Indeed, polymeric compounds were also observed with the fivefold increase in the sorbitol concentration (from 22.4 g·L^−1^ to 112 g·L^−1^). This is an advantage for the one-pot conversion of cellulose, because the concentration of sorbitol produced from cellulose is usually low. The one-pot conversion of cellulose was investigated first using a MCC Avicel PH-101 cellulose (0.8 g), Ru/C (200 mg), water (40 mL), under 5 MPa of initial H_2_ pressure at room temperature. It was shown that fast and selective hydrogenation of glucose is required to increase the isosorbide yield and that at elevated temperatures, there is a selectivity loss through degradation of glucose to insoluble byproducts. Side reactions can be minimized by decreasing the reaction temperature to 210 °C, improving the hydrogenation capacity by optimization of the concentration of Ru/C and decreasing the solvent volume to 40 mL. A formation of isomerization products of isohexides was observed as the isomerization of glucose is acid-catalyzed, and the isomerization of alditols is catalyzed by Ru/C. An isosorbide yield of 52% was obtained after 1 h at 210 °C and 50 bar of H_2_ from 10 wt % of cellulose and a catalyst/cellulose ratio of 1:4 in the presence of 1 g of Ru/C (5 wt % of Ru). It was also shown that a longer reaction time led to an increased production of insoluble byproducts. The productivity of isosorbide can reach 40.2 g·L^−1^·h^−1^with a purity of 73%. The recycling of the metal-supported catalyst (Ru/C) was studied and a high catalytic activity decrease was observed after the first run, showing that the catalyst was not recyclable. Despite their effort to regenerate the catalyst, the loss of activity and yield was always observed. The scope of biomass used was investigated and experiments were performed using hardwood (42% cellulose, 4% hemicellulose and 18% lignin), soft wood (35% cellulose, 23% hemicellulose and 27% lignin), nonpretreated wheat straw (35% cellulose, 1% hemicellulose and 18%lignin) and pretreated wheat straw as raw materials in order to investigate the influence of the delignification on the cellulose conversion. Two pretreatments were used: i) The ethanosolv method that consists in the treatment of wheat straw for 1.5 h in an ethanol/water (50:50) mixture at 210 °C, and ii) CIMV (Compagnie Industrielle de la Matière Végétale) technology, where wheat straw was treated during 3.5 h of acetic acid/formic acid/water (50:30:15 m/m/m) extraction at 105 °C and then in the presence of H_2_O_2_ and organic acids (peracetic and performic acids) for further delignification. After the pretreatment, the cellulose content is 69% and 82% and the lignin content 16% and 8%, respectively. The results obtained from the nontreated biomass showed that nontreated softwood, containing a higher hemicellulose amount compared to hardwood and wheat straw led to the highest isosorbide yield of 7%. This can be due to a higher carbohydrate content and a lower amount of impurities than in the other raw materials. Based on this, the pretreatment of the delignified biomass will promote the synthesis of isosorbide. Hence, CIMV-delignified wheat straw pulp led to 63% of isosorbide, corresponding to a productivity of 40 g·L^−1^·h^−1^ with 72% of purity. This high yield can be ascribed to several parameters such as small particle diameter, large surface area/porosity and lower crystallinity. For the particle size, cellulose fiber diameters are 2 µm and 50 µm of respectively pre-treated wheat straw and microcrystalline Avicel PH-101, that can explain the higher yield of isosorbide from CIMV-delignified wheat straw pulp. The crystallinity and the particle size effects were confirmed by starting from a ball-milled Avicel PH-101 cellulose. A higher yield of isosorbide (around 65%) was observed than from non treated Avicel PH-101 cellulose (52%).

**Scheme 5:**
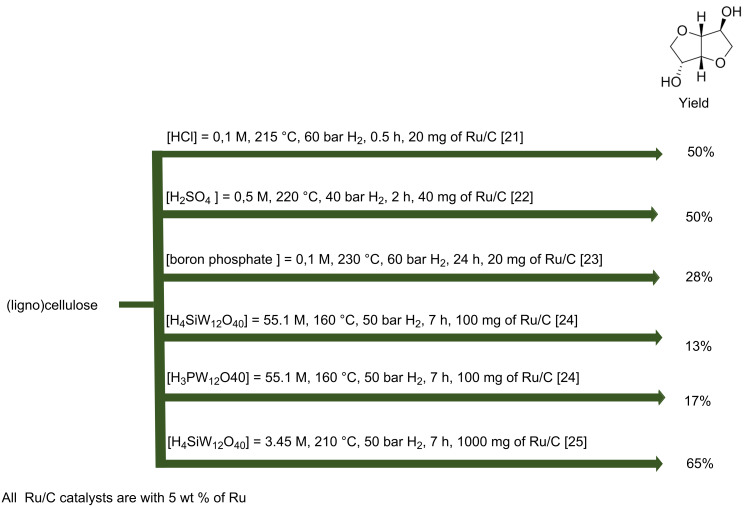
Summary of the results obtained in one-pot one step processes [21–25].

A one-pot conversion of cellulose to isosorbide has been reported using a combination of supported metal catalyst and a homogeneous acid such as mineral acids and heteropolyacids (Scheme 5). The maximum isosorbide yield observed is 65% under optimal conditions. In these processes, a neutralization procedure is essential to remove homogeneous acid catalysts and also separation processes of the products from the salt solutions are required. Regarding an industrial application, if heteropolyacids are used with low concentration, a reduced salt will be formed after the neutralization step. Moreover, heteropolyacids can be precipitated via ion exchange with larger cations, e.g., K^+^, Cs^+^ and NH_4_^+^, or in certain cases even extracted to allow direct recycling. Thus, the usage of heterogeneous acid catalysts is desirable for easy separation of the products. Thus, from a sustainable point of view, it is of interest to study heterogeneous catalysts for the direct conversion of cellulose or lignocellulosic biomass to isosorbide.

### Heterogeneous catalysts for the conversion of (ligno)cellulose to isosorbide

**Scheme 6:**
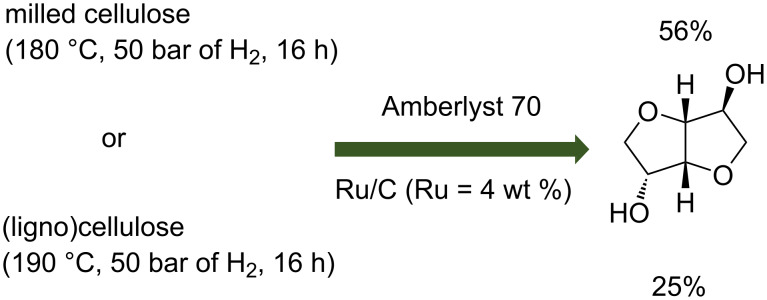
Conversion of (ligno)cellulose to isosorbide in the presence of Amberlyt 70 and a Ru/C catalyst [26–27].

Solid acid catalysts such as ion exchange resins exhibiting sulfonic groups on their external surface resembling to some extent *p*-toluenesulfonic acid (*p*-TSA) can be used in combination with supported metal catalyst (Scheme 6).

Yamaguchi et al. used a combination of Ru/C and Pt/C with Amberlyst 70, an ion-exchange resin containing sulfonic acid sites in the one-pot conversion of ball-milled cellulose to isosorbide [26]. A yield of 8% of isosorbide was obtained at 190 °C in the presence of Pt/C (Pt = 2 wt %) catalyst (0.2 g), 1 g of Amberlyst 70 from 0.324 g of milled cellulose diluted in 40 g of water under 50 bar of H_2_ during 16 h in a batch reactor of 100 mL. This yield was lower than the expected one (38%) using a sequential process. They have shown that the amount of Amberlyst 70 is important and under optimized conditions (0.2 g of 2 wt % Pt/C and 3 g of Amberlyst 70) 16% of isosorbide were obtained. When the loading of Pt was increased from 2 wt % to 4 wt %, 30% of isosorbide was observed at 453 K with 5 MPa H_2_ for 16 h in the presence of 0.3 g of Amberlyst 70. The same approach and reaction conditions were used replacing the Pt/C catalyst by Ru/C (Ru = 4 wt %) catalytic material. Higher isosorbide yields were observed in the presence of the Ru/C catalyst compared to those obtained in the presence of the Pt/C catalyst suggesting that Ru/C is more active for the production of isosorbide from cellulose with Amberlyst 70. Best results are obtained under the following conditions: 0.2 g of 4 wt % Ru/C, 3 g of Amberlyst 70, isosorbide yield of 56% at 180 °C under 50 bar of H_2_ after 16 h of reaction. A comparison between these results and sequential reactions leading in the first step (hydrogenolysis of cellulose) to 51% of sorbitol and in the second step (dehydration of sorbitol) to 63% of isosorbide showed that the activity of the Ru/C catalyst was enhanced by the presence of Amberlyst 70 in the one-pot reaction. On the opposite, the Pt/C activity was inhibited by the presence of Amberlyst 70. The effects of the Ru loadings and catalyst amounts on the isosorbide yield from cellulose were also investigated. An optimum amount of 0.2 g of 4 wt % Ru/C led to 56% of isosorbide whereas 0.4 g of 2% Ru/C led to a lower isosorbide yield (24%) despite the same amount of Ru atoms on these catalysts. The metal dispersion of these two catalysts was almost identical but the particle size differs (2.6 nm for 2% Ru/C and 5.3 nm for 4% Ru/C) suggesting that the Ru species formed in the 4 w. % Ru/C catalyst are more active for conversion of cellulose into isosorbide. The recycling of 4 wt % Ru/C and Amberlyst 70 catalysts was studied at different temperatures. At all the temperatures studied, the isosorbide yield decreased due to carbon deposition on the metal.

Amberlyst 70 was also used in the conversion of lignocellulosic feedstocks to isosorbide in the presence of supported metal catalysts [27]. Japanese cedar, eucalyptus and bagasse are used and they contain between 37% and 41% of cellulose and a glucose content between 40.1% and 46.5%. The biomass feedstock was crushed by a simple ball milling procedure without further treatment. The one-pot conversion of Japanese cedar was carried out by combining Pt/C or Ru/C with 4 wt % of metal and Amberlyst 70 catalysts in a batch reactor of 100 mL. Ru/C catalyst was more active than Pt/C catalyst. Indeed, in the presence of Ru/C (0.2 g) and Pt/C (0.3 g) catalysts, an isosorbide yield of 25% and 2% was obtained, respectively, at 190 °C under 50 bar of H_2_ after 16 h in the presence of 3 g of Amberlyst 70. They supposed that the Ru/C catalyst exhibits a better activity owing to Amberlyst 70 as shown in their previous work [22]. A one-pot conversion with Amberlyst 70 and Ru/C is thereafter tested in eucalyptus and bagasse feedstocks. Interestingly, 8% and 13% of isosorbide were observed from eucalyptus and bagasse respectively at 190 °C under 50 bar of H_2_ after 16 h in the presence of 3 g of Amberlyst 70 and 0.2 g of Ru/C. They suggest that lignin or an inorganic component contained in eucalyptus and bagasse can affect the acidity of Amberlyst 70 which implies the lower yield of isosorbide and the higher yield of 1,4-sorbitan.

Xi et al. studied a combination of a Ru/C catalyst with mesoporous niobium phosphate and Ru support over mesoporous niobium phosphate in the conversion of cellulose to isosorbide [28]. Different preparation methods of NbOPO_4_ were performed depending on the final pH (2, 7 and 10) of the solution at the end of the solid synthesis. Based on the results obtained in the dehydration of sorbitol to isosorbide, the NbOPO_4_-pH_2_ catalyst was chosen for the production of isosorbide from cellulose. Two strategies were employed: a one-step and a two-step process. The one-step conversion of cellulose to isosorbide consists in using the same reaction conditions and the same catalyst for the hydrolysis/hydrogenation and dehydration reactions. An isosorbide yield of 13% was obtained in the presence of 5% Ru/NbOPO_4_-pH_2_ (0.2 g) at 230 °C and 24 h of reaction in a 100 mL stainless steel reactor starting from cellulose (0.24 g) in 15 mL of water. In the case of an association of Ru/C and NbOPO_4_-pH_2_, the yield of isosorbide was around 20%. The one-step strategy seems to be not efficient for the conversion of cellulose to isosorbide due to the temperature of the reaction that is different for the two reactions (hydrolysis/hydrogenation and dehydration). To overcome this temperature issue a two-step process was studied. In the first step, 0.24 g of cellulose was added in 15 g of water at 170 °C for 24 h in the presence of 0.1 g of Ru/NbOPO_4_-pH_2_. Ru/NbOPO_4_-pH_2_ was then removed and the aqueous solution containing sorbitol and sorbitans was dehydrated at 230 °C during 18 h in the presence of 0.1 g of NbOPO_4_-pH_2_ for the second step. After the first step, an aqueous mixture of 59% of sorbitol, 20% of 1,4- and 3,6-sorbitans, respectively, 4% of 2,5-sorbitan and 4% of isosorbide was obtained. The second step led to an isosorbide yield of 56% which is higher than the yield obtained after the one-step strategy. The two-step reaction was also performed using a similar Ru/NbOPO_4_-pH_2_ catalyst in both steps and the yield of isosorbide was 33%. This decrease was due to a different acidity. Hence, the ruthenium species occupied the strong and medium acid site of NbOPO_4_-pH_2_ after its impregnation. The recyclability of NbOPO_4_-pH_2_ was performed for the two-step conversion of cellulose to isosorbide as the Ru/NbOPO_4_-pH_2_ recyclability was proved in a previous study in the conversion of cellulose to isosorbide [29]. A decrease of approximately 10% in isosorbide yield is reported after four runs due to carbon deposition (confirmed by thermogravimetry analysis) or during the recycling procedure. However, the carbon deposition was removed by calcination of the catalyst at 400 °C and a similar isosorbide yield as the one obtained at the first run was observed.

**Scheme 7:**
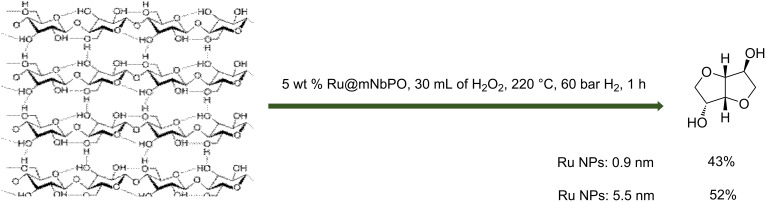
Use of Ru-supported on mesoporous nobium phosphate (mNbPO) for the synthesis of isosorbide from cellulose [30].

A sustainable method for the isosorbide production from cellulose is the use of a bifunctional catalyst (Scheme 7). A series of Ru catalysts on acid support were prepared by the adsorption of colloidal Ru nanoparticles on mesoporous and bulk niobium phosphate, mesoporous and bulk niobium oxide hydrate, phosphoric acid-treated mesoporous and bulk niobium oxide hydrate [30]. These catalysts were compared to Ru@HZMS5, Ru@NaY, Ru@γ-Al_2_O_3_ solids. Cellulose was converted over all niobia-based catalysts with 25–43% yield of isosorbide starting from 0.6 g cellulose using 0.06 g catalyst (5.0 wt % Ru, Ru nanoparticles (NPs): 0.9 nm) in 30 mL H_2_O after 1 h of reaction at 220 °C under 60 bar of H_2_. Trace amounts or even no isosorbide was obtained over microporous HZSM-5, NaY, and γ-Al_2_O_3_-supported Ru catalysts. A bifunctional Ru catalyst supported on mesoporous niobium phosphate with a mean size of Ru NPs of 5.5 nm was used for the direct conversion of cellulose to isosorbide and 52% yield of isosorbide was observed with almost 100% cellulose conversion. The large surface area, pore size, and strong acidity of mesoporous niobium phosphate are key parameters for the hydrolysis of cellulose and dehydration of sorbitol. An appropriate size of the supported Ru nanoparticles avoids unnecessary hydrogenolysis of sorbitol. The recyclability was investigated and up to 6 cycles could be performed without significant loss in catalytic properties. Moreover, no leaching of Ru was observed. This study shows that a bifunctional catalyst can be a more sustainable solution to produce isosorbide from (ligno)cellulose.

## Conclusion

The isosorbide synthesis from cellulose is performed via acid-catalyzed hydrolysis of cellulose, followed by hydrogenation of glucose to sorbitol and further dehydration to sorbitans. The dehydration of sorbitol to isosorbide required temperatures around 200 °C exposing hydrogenation catalysts to the harsh reaction conditions in the one-pot process. Unfortunately, one-step conditions seem to facilitate the deactivation of the hydrogenation catalysts, thus preventing their efficient recycling due to the production of insoluble byproducts, metal particle sintering and leaching. From all the studies, it is clear that Ru/C was the most active catalyst in the hydrogenation reaction. Based on the stability of the hydrogenated catalyst, some studies used a two-step process to increase the isosorbide yield. One important parameter is also the acidity strengths of the acid catalyst used. An appropriate acidity is required to depolymerize cellulose to glucose and to dehydrate sorbitol to isosorbide. If the acidity is too high, byproducts are formed decreasing the yield of isosorbide. Another important factor is the nature of the feedstocks used. Hence, most of the isosorbide syntheses are from pure cellulose and sugars as substrates, such as microcrystalline cellulose and glucose, while studies describing an isosorbide synthesis from lignin-containing cellulosic substrates are scarce. The presence of lignin can be detrimental for the activity of the catalysts. Hence, investigations of reaction conditions and the development of the hydrogenation catalysts to tolerate the presence of lignin should be further investigated.

For the one-pot conversion of lignocellulose to isosorbide, a sustainable and interesting route should be the design of a heterogeneous catalyst composed of a metal supported on an acid support. These catalysts should be designed in order to be stable in water and tolerant to the presence of lignin. Otherwise, the solution should be to realize this reaction under continuous flow using two catalytic beds.

Finally, we emphasize that mechanistic insights on the catalytic conversion of biomass-derived platform molecules are required to make this value chain competitive with respect to the traditional synthesis of chemicals from fossil fuels. In particular, in situ kinetic studies by spectroscopic methods [31] correlated to computational chemistry approaches [32] might reveal specific interaction between reactants and catalysts as well as solvent effects at work in such a complex system that should provide an understanding of the underlying reason for the observed unique kinetics, yields and selectivity in these one-pot reactions. In addition, such in-depth fundamental mechanistic and kinetic studies should enable determining the key structural parameters of the catalytic platform that govern its efficiency hence supporting the design of novel highly active and selective catalysts for the valorization of cellulose as a sustainable feedstock.
